# The issue of heterogeneity of MSC-based advanced therapy medicinal products–a review

**DOI:** 10.3389/fcell.2024.1400347

**Published:** 2024-07-26

**Authors:** Ana Bajc Česnik, Urban Švajger

**Affiliations:** ^1^ Slovenian Institute for Transfusion Medicine, Department for Therapeutic Services, Ljubljana, Slovenia; ^2^ Faculty of Pharmacy, University of Ljubljana, Ljubljana, Slovenia

**Keywords:** mesenchymal stromal (stem) cell, heterogeneity, pooling, equipotency, advanced therapy medicinal product (ATMP), cell therapy

## Abstract

Mesenchymal stromal stem cells (MSCs) possess a remarkable potential for numerous clinical applications due to their unique properties including self-renewal, immunomodulation, paracrine actions and multilineage differentiation. However, the translation of MSC-based Advanced Therapy Medicinal Products (ATMPs) into the clinic has frequently met with inconsistent outcomes. One of the suspected reasons for this issue is the inherent and extensive variability that exists among such ATMPs, which makes the interpretation of their clinical efficacy difficult to assess, as well as to compare the results of various studies. This variability stems from numerous reasons including differences in tissue sources, donor attributes, variances in manufacturing protocols, as well as modes of administration. MSCs can be isolated from various tissues including bone marrow, umbilical cord, adipose tissue and others, each with its unique phenotypic and functional characteristics. While MSCs from different sources do share common features, they also exhibit distinct gene expression profiles and functional properites. Donor-specific factors such as age, sex, body mass index, and underlying health conditions can influence MSC phenotype, morphology, differentiation potential and function. Moreover, variations in preparation of MSC products introduces additional heterogeneity as a result of cell culture media composition, presence or absence of added growth factors, use of different serum supplements and culturing techniques. Once MSC products are formulated, storage protocols play a pivotal role in its efficacy. Factors that affect cell viability include cell concentration, delivery solution and importantly, post-thawing protocols where applicable. Ensuing, differences in administration protocols can critically affect the distribution and functionallity of administered cells. As MSC-based therapies continue to advance through numerous clinical trials, implication of strategies to reduce product heterogeneity is imperative. Central to addressing these challenges is the need for precise prediction of clinical responses, which require well-defined MSC populations and harmonized assessment of their specific functions. By addressing these issues by meaningful approaches, such as, e.g., MSC pooling, the field can overcome barriers to advance towards more consistent and effective MSC-based therapies.

## 1 Introduction

**FIGURE 1:**
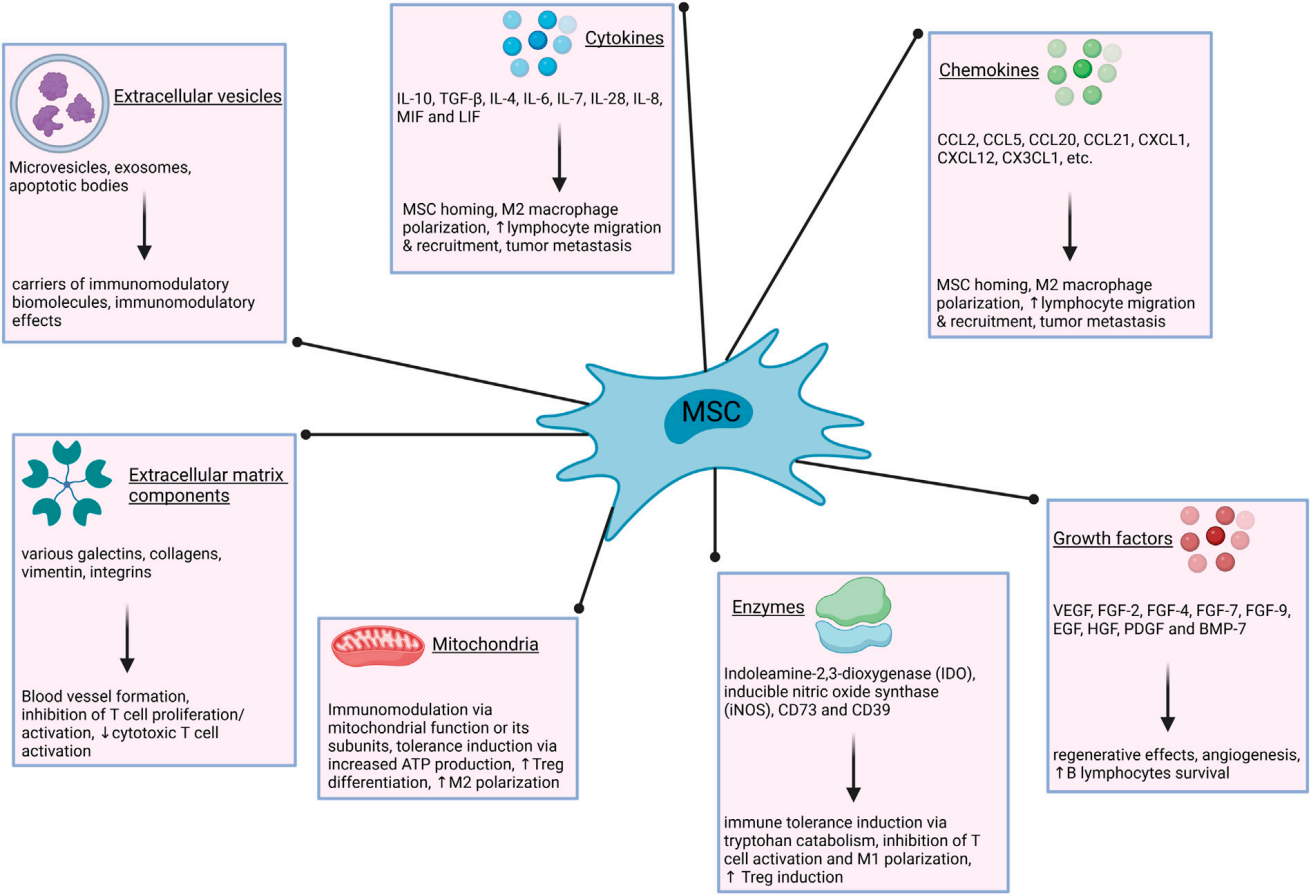
Immunomodulatory mechanisms of MSCs. The modulation of immune responses by MSCs is exerted via numerous secreted factors and entities, such as cytokines, growth factors, extracellular vesicles and others. ATP–adenosine triphosphate; BMP–bone morphogenetic protein; CCL–chemokine (C-C motif) ligand; CX3CL–chemokine (C-X3-C motif) ligand; CXCL–chemokine (C-X-C motif) ligand; EGF–epidermal growth factor; FGF–fibroblast growth factor; HGF–hepatocyte growth factor; IL–interleukin; LIF–leukemia inhibitory factor; M1–type one macrophages; M2–type two macrophages; MIF–macrophage migration inhibitory factor; PDGF–platelet derived growth factor; TGF–transforming growth factor; VEGF–vascular endothelial growth factor.

Mesenchymal stromal cells (MSCs) are a heterogeneous population of somatic stem cells with a capacity for self-renewal, multilineage differentiation, and immunomodulation (Figure 1). They are considered a promising therapeutic tool to control aberrant inflammatory responses and assist in regenerative medicine applications as Advanced Therapy Medicinal Products (ATMPs) (Cheung et al., 2020; Maldonado et al., 2023). Very briefly, according to EU legislation and classification, ATMPs are defined as medicines for human use that are based on genes, tissues or cells. The have been classified into three main types, namely, gene therapy medicinces, somatic-cell therapy medicines and tissue-engineered medicines. According to this classification, MSC-based ATMPs are somatic-cell therapy medicines, and their therapeutic use has been studied for a broad range of diseases. Some of the conditions proposed to benefit from MSC treatment are graft-versus-host-disease (GvHD) (Kelly and Rasko, 2021; Kadri et al., 2023), Crohn’s disease (Wang et al., 2023), critical limb ischemia (Lozano Navarro et al., 2022), osteoarthritis (Thoene et al., 2023), type 1 diabetes (Koehler et al., 2022), type 2 diabetes (Gao et al., 2022), endometrial injury (Cen et al., 2022), multiple sclerosis (Liu et al., 2022), lupus (Li et al., 2013), cardiovascular diseases (Mabotuwana et al., 2022), liver disorders (Han et al., 2022), respiratory disorders (Raza and Khan, 2022), spinal cord injury (Montoto-Meijide et al., 2023), kidney failure (Morello et al., 2022), skin diseases (Chang et al., 2021; Lwin et al., 2021), Alzheimer’s disease (Regmi et al., 2022), and Parkinson’s disease (Kouchakian et al., 2021). Administration of MSCs continuously proves to be safe with very little evidence of serious adverse events such as infusion-related toxicity, infection, malignancy and development of thrombotic or thrombo-embolic events (Thompson et al., 2020; Wang Y. et al., 2021), athough exceptions have been observed (Veceric-Haler et al., 2022).

A plethora of investigations involving MSC products shows their preclinical and early clinical efficacy can be inconsistent and remains frequently unconfirmed in late-phase trials. It can also be challenging to anticipate, as most of the *in vitro* assays have failed to reproducibly and reliably predict the clinical potency of transplanted MSCs (Krampera and Le Blanc, 2021). At least in part, the inconsistencies of these outcomes could be attributed to the heterogeneity of transplanted MSC batches. The first important step toward greater harmonization was made in 2006, when basic criteria for MSC characterization have been proposed by the International Society for Cell and Gene Therapy (ISCT), and are as follows:

• adherence to plastic in standard culture conditions,
• specific surface antigen expression (≥95% of the MSC population must express CD105, CD73 and CD90, and lack expression (≤2% positive) of CD45, CD34, CD14 or CD11b, CD79a or CD19 and HLA class II as measured by flow cytometry),
• multipotent differentiation potential–they must be able to differentiate into osteoblasts, adipocytes and chondroblasts under standard *in vitro* differentiating conditions. (Dominici et al., 2006).

However, a scoping review by Renesme et al. reports that only 18% of randomly analyzed studies involving MSC explicitly referred to the ISCT criteria. More precisely, only 36% of the studies reported plastic adherence, 40% reported any kind of *in vitro* differentiation assay and 53% of the studies performed analysis of cell markers (Renesme et al., 2022). Since MSC-based products are regarded as medicinal products according to EU legislation, it is of further importance particularly for future studies, that uniformity of their characteristics and efficacy is comprehensible and unambigous to the greatest possible extent. In this review, we take a closer look at the origins of MSC variability, their impact on clinical and preclinical studies, and propose potential solutions to address these issues.

## 2 Origins of MSC heterogeneity

ISCT acknowledges that MSCs encompass a heterogeneous population pool, which includes fibroblasts, myofibroblasts, and a small proportion of stem/progenitor cells, while lacking hematopoietic or endothelial cells (Viswanathan et al., 2019). Single-cell RNA sequencing (scRNA-seq) of MSCs has identified several candidate subpopulations with different functional characteristics - some exhibit greater proliferation ability while others show higher osteogenic, chondrogenic or adipogenic differentiation potency and maintenance of stemness (Sun et al., 2020; Wang et al., 2021b; Hou et al., 2021; Chen et al., 2022; Xie et al., 2022). It has been shown that extracellular matrix highly contributes to the heterogeneity of MSC populations in a tissue-type-dependent pattern (Wang et al., 2021c). The secretory and immunomodulatory functions linked to clinical benefits in MSC-based therapies are believed to arise from the bulk, heterogeneous stromal cell fraction (Viswanathan et al., 2019). However, others argue that different MSC populations should be separated immediately after isolation, individually expanded *in vitro* and selected according to their characteristics to treat different diseases (Wang et al., 2017; Zhang S. et al., 2021). Still, research shows that even colonies originating from a single cell will in time become functionally heterogeneous (Rennerfeldt et al., 2019).

**FIGURE 2:**
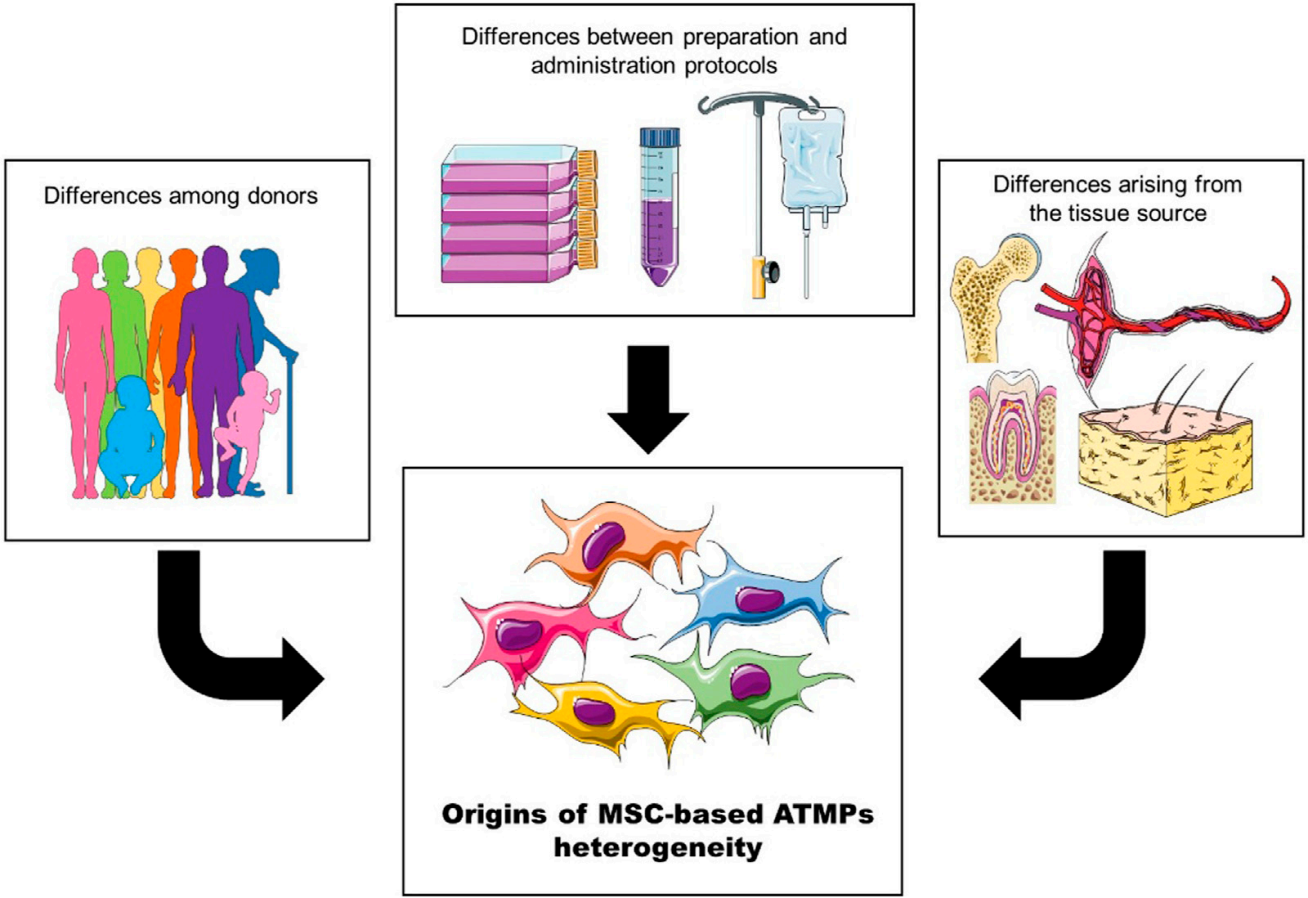
The heterogeneity of MSC-based ATMPs can be broadly categorized into three groups: differences among donors, variations arising from the tissue source, and differences introduced by preparation and administration protocols.

Due to the variations in multiple factors across studies, pinpointing key aspects that influence clinical outcomes can be challenging. However, they can be broadly categorized into three groups (Figure 2):

• Differences arising from the tissue source,
• Donor attributes,
• Preparation and administration protocols.

### 2.1 Heterogeneity arising from tissue source

Throughout the years, the acronym MSC has accumulated quite a bit of controversy, starting with Caplan, who in 1991 coined the term adult “mesenchymal stem cells”, referring to a small number of cells involved in repair and turnover of skeletal tissues (Caplan, 1991). Later, similar cells have been found in most anatomical locations and researchers called for a name change from mesenchymal stem cells to mesenchymal stromal cells, reflecting their stromal residence (Dominici et al., 2006). However, disputes continue, as some would change the term to multipotent stromal cells, while others go as far as to argue for complete abolition of the acronym MSC (Sipp et al., 2018; Soliman et al., 2021). Currently, the official position of the International Society for Cell and Gene Therapy (ISCT) Mesenchymal Stromal Cell committee is that the acronym “MSC” should remain in use, however, information about the tissue source should always be provided (Viswanathan et al., 2019).

MSCs reside and can be isolated from various tissues, including bone marrow (BM-MSCs) (Li et al., 2016), umbilical cord blood (CB-MSCs) (Um et al., 2020), umbilical cord tissue (UC-MSCs), Wharton’s jelly (WJ-MSC) and fetal placenta (Beeravolu et al., 2017), adipose tissue (AD-MSCs) (Ong et al., 2021), dental tissue and dental pulp (DT-MSCs and DP-MSCs) (Li et al., 2023), fetal liver (Gridelli et al., 2012), endometrial tissue and menstrual blood (Meng et al., 2007; Allickson et al., 2011; Schüring et al., 2011; Bozorgmehr et al., 2020). Although the majority of research focuses on BM-MSCs, AD-MSCs and UC-MSCs (as well as WJ-MSCs), MSCs from other tissues have noticeable therapeutic benefits. For instance, DP-MSCs also possess the capacity to differentiate into different cell types and are quite extensively used in regenerative medicine, although mainly in preclinical research. Thus, they have been used for tissue repair in context of periodontal diseases, tooth reconstruction, dental pulp regeneration, as well as for distant anatomical tissues, e.g., for regeneration of neuronal and skeletal tissue damage (Graziano et al., 2008; Ledesma-Martinez et al., 2016). Nevertheless, the most commonly used sources are bone marrow, followed by umbilical cord and adipose tissue (Naji et al., 2019).

MSCs isolated from different sources share many common characteristics, but they also show particular phenotypic and functional differences (Patel et al., 2016; Wu et al., 2018; Rady et al., 2020a; Shin et al., 2021; Li Y. et al., 2022; Li S. et al., 2022; Laloze et al., 2023). Comparison of scRNA-seq of BM-MSC and UC-MSC has revealed more differences in gene expression between tissue sources than between individual donors (Medrano-Trochez et al., 2021). Moreover, a massive parallel multiplexing scRNA-seq performed across multiple tissues and donors has revealed a tissue-type-dependent pattern of MSC subpopulations, indicating that MSCs from different tissues have prominent transcriptomic heterogeneity (Wang et al., 2021c). This also gave rise to the rationale that patients burdened with certain pathologies could benefit from MSCs sourced from a specific tissue, which could be functionally relevant for their clinical efficacy.

Still, MSC capabilities (and identity) from different tissues are not fully characterized, leading to contradictory results. For example, under the same culturing conditions, AD-MSCs displayed the highest immunosuppressive potency, followed by BM-MSCs and UC-MSCs (Calcat-i-Cervera et al., 2023). Others report BM-MSCs to have the lowest immunosuppressive abilities compared to AD- and UC-MSCs (Ketterl et al., 2015a). On the other hand, BM-MSCs showed a superior capacity to support angiogenesis and induce endothelial cell migration in comparison to AD-MSCs or UC-MSCs (Calcat-i-Cervera et al., 2023). Similarly, comparing gene expressions of BM-MSCs and AD-MSCs from the same pool of donors has revealed distinct transcriptomic profiles that directly translate into MSC capacity to interact with immune cells (Ménard et al., 2019). For example, the study showed that BM-MSCs were better at suppressing NK cell proliferation, while AD-MSCs were better at suppressing T cell proliferation.

### 2.2 Donor heterogeneity

Another factor contributing to the difficulties in standardization of MSC products is the high variability between donors, which includes a multitude of factors, such as donor age, sex, BMI, as well as systemic and autoimmune diseases (Sun et al., 2007; Siegel et al., 2013; Patel et al., 2016; Rady et al., 2020b). Such variabilities can manifest as differences in MSC phenotype, morphology, doubling time, immunosuppressive potential, gene expression, proliferation, differentiation, and colony-forming capacity (CFU) (Siegel et al., 2013; Ganguly et al., 2019; Li S. et al., 2022). For instance, AD-MSCs from older donors have shown increased cellular senescence, reduced viability and proliferation, as well as reduced differentiation potential in comparison to younger donors (Choudhery et al., 2014). Similarly, BM-MSCs from infant donors doubled more quickly, differentiated into bone and fat cells more efficiently and formed more and denser CFUs. They were also better at suppressing T cell proliferation at lower concentrations than BM-MSCs from adult donors (Myneni et al., 2019). Furthermore, single-cell multiomic analysis profiling the transcriptome and epigenome of BM-MSCs from four healthy donors allowed for classification of cells into four clusters, indicating that BM-MSCs from different donors possess distinct chromatin accessible regulatory elements, which was reflected as variations in their differentiation potential into osteoblasts (Chen et al., 2023). Interestingly, it has been shown that MSCs’ immunomodulatory and angiogenic fitness are inversely correlated and can predict inter-donor differences in proangiogenic *versus* anti-inflammatory/immune suppressive activities in cell-based assays (Robb et al., 2022a; Lee et al., 2023).

It would be rational to assume that deriving MSCs from umbilical cords would eliminate certain heterogeneity originating from donors’ age, lifestyle, and pathophysiological conditions. Remarkably, an opposing trend has been observed. A study examining the cellular heterogeneity in single-cell transcriptomes of MSCs discovered higher inter-donor variability in WJ-MSC samples compared to BM-MSC samples (Zhang C. et al., 2022). In another scRNA-seq study, UC-MSCs exhibited significantly higher heterogeneity in their subpopulations across different donors when compared to MSCs from adult donors (Wang et al., 2021d).

Moreover, in a study comparing MSCs derived from umbilical cords of 12 donors, doubling time and population doubling varied by a factor of two between donors (Mebarki et al., 2021). A comparison of UC-MSCs from 32 donors revealed a substantial variability in both their proliferation rates and immunomodulatory properties (Zhang C. et al., 2021). Interestingly, while no correlation was found between their proliferation rates and immunosuppressive capacity, the latter exhibited a close alignment with their therapeutic effects observed in a mouse spinal cord injury model (Zhang C. et al., 2021; Zhu et al., 2022). In a study aimed at standardizing isolation and expansion methods, MSCs derived from umbilical cords of 90 donors were examined. Interestingly, lower gestational age was associated with a shorter time to P0 harvest, suggesting that even minor variables, such as time of delivery, could potentially exert a significant influence on MSC characteristics (Todtenhaupt et al., 2023).

A comparison of CB-MSCs from seven donors identified two distinct groups based on angiogenic capacity under hypoxic conditions: one with low and another with high angiogenic potential. These distinctions correlated with the differential expression of four specific genes—ANGPTL4, ADM, CDON, and GLUT3—which were chosen based on prior research highlighting their roles in angiogenesis and sensitivity to hypoxic conditions (Kang et al., 2018a).

The discussed donor variability stands as a significant barrier in the development of consistent protocols and cellular medicinal products. Despite efforts to control for variables with substantial impacts on the clinical quality of MSCs by using more primitive MSCs, such as UC- and less often CB-MSCs, heterogeneity between batches clearly persists. Exploring strategies like, e.g., pooling cells from different donors might perhaps mitigate these effects and ensure more comparable products, leading to more consistent results.

### 2.3 Heterogeneity introduced by variations in preparation and administration protocols

Clinical efficacy of MSC products can vary considerably, depending not only on tissue source and donor characteristics but also on preparation and administration protocols. Given the regularly irreproducible effectiveness of MSCs in clinical trials, the optimization of cell manufacturing protocols is still a work in progress. However, this pursuit also holds the potential to further introduce heterogeneity into preparation conditions. Despite advancements towards standardization of the production procedures and accurate characterization of the MSC products, variabilities among manufacturing centers are very much present (Bieback et al., 2019). Understanding these differences and their impact on product quality would allow for a better comparison of the clinical outcomes across various institutions.

#### 2.3.1 Media supplements

Efficient MSC expansion in culture requires basal medium supplemented with growth factors, proteins, and enzymes to support attachment, growth, and proliferation. The most commonly used supplement in cell culture media in general is fetal bovine serum (FBS), due to its rich supply of growth factors, cytokines, and chemokines (Bieback, 2013).

However, the utilization of FBS in cell culture poses scientific, economic and moral issues (Subbiahanadar Chelladurai et al., 2021). The most critical concern in using FBS for clinical applications is its potential contamination with xenogeneic elements, including prion proteins, endotoxins, various types of microbes, immunoglobulins, and viruses. Another issue pertains to the uncertainty surrounding the precise composition of FBS and its batch-to-batch variability, both of which can impact the biological properties of cultured cells. Thirdly, the growing demand and limited production capacity can result in unpredictable shortages and higher prices of FBS. Last but not least, the increasing number of fetuses slaughtered specifically for FBS production and the potential fetal distress during blood collection give rise to significant ethical concerns regarding animal welfare (Subbiahanadar Chelladurai et al., 2021).

To address these issues, alternatives to FBS are being developed and integrated into MSC manufacturing protocols. Among the most widely adopted alternatives are human platelet lysate (Cañas-Arboleda et al., 2020), pooled human AB-serum (Savelli et al., 2018), human umbilical cord serum (Afzal et al., 2023), and serum-free media (Caneparo et al., 2022). Nevertheless, similar to FBS, these alternatives struggle with certain challenges. For example, platelet lysate-plasma contains fibrinogen and other coagulation factors. To prevent gelation, commercially available platelet lysate usually contains animally-sourced heparin, making it no longer xeno-free (Altrock et al., 2023). Supplements derived from human blood also share some concerns with FBS, particularly regarding the potential transmission of infectious agents and the variability in their composition (Bieback, 2013).

Culturing MSCs in variously supplemented media can lead to alterations in their fundamental characteristics, including changes in proliferation rate, morphology, gene expression patterns, senescence, immunomodulatory properties, and differentiation capacity (Dam et al., 2021; Takao et al., 2021; Jakl et al., 2023). It has been shown that growing MSCs in serum-free media leads to smaller cells with increased proliferation rate that are better at forming colonies than those grown in FBS-supplemented media (Aussel et al., 2022; Caneparo et al., 2022). However, they exhibited lower osteogenic and chondrogenic differentiation capacity (Caneparo et al., 2022).

Similarly, BM-MSCs grown in medium with human platelet lysate showed faster proliferation rates and lower differentiation capacity compared to those in FBS-supplemented medium (Anerillas et al., 2023). Additionally, BM-MSCs expanded in medium with human AB-serum exhibited round cell enrichment, better adhesion, and faster proliferation rates compared to those in medium with human platelet lysate (Savelli et al., 2018). In a study comparing human platelet lysate, fetal bovine serum, and human AB-serum, platelet lysate emerged as the superior choice for supporting AD-MSC proliferation, differentiation, and growth in 3D cultures (Kirsch et al., 2021).

Considering the collective body of research, determining the ideal supplement for MSC manufacturing remains a challenging task. However, although MSCs cultured in media supplemented with platelet lysate exhibit notably accelerated proliferation and marked variations in cellular morphology compared to those cultured in FBS, no discernible alterations in DNA-methylation patterns have been observed, and only modest differences in gene expression profiles were detected. Moreover, the changes in proliferation and morphology proved to be reversible (Fernandez-Rebollo et al., 2017).

#### 2.3.2 Culturing techniques

While one of the defining criteria for MSCs has been *in vitro* plastic adherence, conventional methods of their extensive 2D *in vitro* expansion are not representative of the *in vivo* environment. Instead, MSCs exist within their niches as a part of heterogeneous cell population, where they tightly adhere to each other and exhibit complex cell-cell and cell-extracellular matrix interactions (Yen et al., 2023).

Conventional MSC manufacturing techniques, selected for their convenience and low cost of implementation, are aimed at generating a clinically relevant number of cells. However, they can negatively impact MSC characteristics and functions, which could be responsible for their limited therapeutic efficacy. In an effort to preserve or enhance MSC phenotypes and consequently improve their *in vivo* performance, 3D culturing techniques have been developed (Kouroupis and Correa, 2021). When grown suspended in culture, MSCs spontaneously coalesce and form spherical multicellular aggregates, termed spheroids (Fuentes et al., 2022). These are thought to better recapitulate *in vivo* interactions, promote secretion of paracrine factors, improve cell survival, increase MSC differentiation potential, and delay their replicative senescence (Cesarz and Tamama, 2016; Yen et al., 2023).

The methods used to generate MSC spheroids can be generally classified as scaffold-free and scaffold-based culture platforms (Kouroupis and Correa, 2021). Scaffold-free methods can be further divided into static and dynamic approaches. The most trivial static technique is growing cells in a non- or low-adherent environment that allows self-organization of cells into suspended spheroids (Redondo-Castro et al., 2018). More complex methods encompass hanging-drop method (Bartosh and Ylostalo, 2014; Au - Ylostalo et al., 2017), forced aggregation (Rettinger et al., 2014) and magnetic levitation (Lewis et al., 2017; Gaitán-Salvatella et al., 2023). Among the most investigated dynamic approaches are spinner flask culture and rotating wall vessel techniques (Marques et al., 2023). Additionally, various scaffold-based MSC spheroid generation methods have been proposed using both natural and synthetic biomaterials. Biomaterial selection should be based on the therapeutic application in mind, as physical-chemical characteristics of scaffolds such as porosity and biodegradation can dramatically affect MSC stemness and differentiation capacities (Kouroupis and Correa, 2021).

Studies show that mild hypoxia present within the inner zones of MSC spheroids may positively affect MSC survival and secretory capacity. 3D culture conditions significantly increase the relative expression of stemness-related transcriptional factors in MSCs and promote their anti-inflammatory profile (Bartosh et al., 2010a; Rybkowska et al., 2023). RNA-seq data obtained from human amnion-derived MSCs following 3D culture revealed increased expression of pleiotropic factors important in tissue regeneration, such as CXCL12, LIF, VEGF-A, HGF, BDNF, IL6, EGF, PGE2, CCL20, BMP2, TGFB1, CXCL1, CCL2, GDF15, IL11, and CCL7 (Gallo et al., 2022). Growing MSCs under hypoxic conditions does not change their specific surface antigen expression (Kang et al., 2018a; Tomecka et al., 2021). Nevertheless, they exhibit improved abilities for multi-lineage differentiation, survival, migration and proliferation, better support of angiogenesis and increased expression of stemness-related genes (Kang et al., 2018b; Meng et al., 2018; Wang et al., 2022). They were also shown to spontaneously generate 3D niche-like structures of undifferentiated, small, round Oct4 and HIF-2a positive fast growing cells (Drela et al., 2014).

As of today, no clinical trials have been conducted to evaluate the therapeutic potential of MSC spheroids. Consequently, there are no specific criteria in place to define conditions where MSC spheroids might be preferred over MSCs expanded in a monolayer. Still, it has become more and more evident that conventional culturing methods cannot ensure the preservation of MSC characteristics and their associated functionality to the same extent. The adoption of reproducible, high-throughput methods that meet regulatory requirements for MSC spheroid production could facilitate their clinical use and potentially lead to MSC products with improved therapeutic efficacy.

#### 2.3.3 Expansion level

MSC-based therapies require a substantial number of cells, as individual doses are measured in millions of cells per kilogram of body mass, particularly for systemic treatments like in GvHD (Kelly and Rasko, 2021). Consequently, to obtain the necessary cell quantity for clinical applications, extensive MSC expansion is inevitable. Unfortunately, such expansion can significantly alter MSC characteristics and has been proposed as a possible cause for poor performance in certain clinical trials (Galipeau, 2013; Hoch and Leach, 2014). These changes can impact phenotypic, morphological, genetic and functional attributes of MSCs, along with their regenerative and immunomodulatory secretome profile (Yang et al., 2018; Miclau et al., 2023).

Indeed, population doubling has been reported to inversely correlate with MSC potency, with early-passage cells being more potent than batches of extensively expanded cells, possibly due to cell senescence (von Bahr et al., 2012). Conversely, there is a theory that rapidly dividing clones with less favorable characteristics may outcompete slower proliferating cells during each passage, gradually increasing the ratio of poorly performing cells. An interesting study by Selich et al. has demonstrated, that when MSCs are first introduced into culture, they constitute a heterogeneous cell population. However, with successive passaging, this initial diversity diminishes, leading to the selection of a limited number of clones in later passages (Selich et al., 2016).

It appears as though expansion of MSCs with optimal function is limited to a few passages, increasing the cost and reducing the feasibility of mass production for MSC therapeutics. Nevertheless, some argue that a brief period of culturing in a 3D format prior to administration could induce extensively expanded MSCs to express and secrete anti-inflammatory and immunomodulatory factors, thereby enhancing their ability to generate a larger cell population (Bartosh et al., 2010b). Further research is required to fully confirm this hypothesis.

#### 2.3.4 Administration protocols

MSCs can be introduced through either systemic or local delivery methods. However, following intravenous infusion, most MSCs get entrapped in pulmonary vasculature, where they form emboli. In about 24h, the vast majority of cells are cleared from the lungs and only a minor fraction home to different organs such as heart, brain, liver and kidney (Lee et al., 2009; Eggenhofer et al., 2014; Luk et al., 2016). To prevent their entrapment in the lungs, MSCs can be administered directly to the site of the lesion or inflammation (Moon et al., 2019; Lamo-Espinosa et al., 2020; Czarnecka et al., 2021; Ouboter et al., 2023). Nevertheless, local administration can be invasive, more complex, and requires additional training for medical practitioners. Moreover, in certain conditions such as GvHD and solid organ transplantation, MSCs cannot be administered locally and instead require systemic delivery (Franquesa et al., 2013; Podesta et al., 2020).

Multiple factors can affect viability and key functional characteristics of MSCs at the time of administration, including cell concentration, the choice of solution in which the cells are delivered and post-thawing protocols (Zhang et al., 2017; Aabling et al., 2023). A droplet-based scRNA-seq comparing pre-freeze and post-freeze BM-MSC samples has identified numerous differentially expressed genes associated with a wide range of cellular functions, such as cytokine signaling, cell proliferation, cell adhesion, cholesterol/steroid biosynthesis, and regulation of apoptosis (Medrano-Trochez et al., 2021). Indeed, in the first 24 h after thawing, cryopreservation reduces cell viability, increases apoptosis level and impairs MSC metabolic activity, immunosuppressive potency and adhesion potential (Ketterl et al., 2015b; Bahsoun et al., 2020; Giri and Galipeau, 2020).

Prior to infusion, cells are usually formulated with a saline solution, human albumin solution or even administered directly in their cryopreservant solution (Trento et al., 2018). It is worth noting that only a limited number of clinical trials specify the handling of MSC products, from dose preparation to cell administration (Wiese et al., 2022).

## 3 Strategies for MSC standardization

Given the numerous ongoing clinical trials involving MSCs and the growing need for large-scale manufacturing protocols, it is imperative to establish consensus assays for MSC processing and the release of MSC products. Reference materials and validated, uniformly applied tests for quality control of MSC products are required (Robb et al., 2019). However, some stakeholders in the MSC field advocate for caution when establishing definitive cell standards, since there are still gaps in our current understanding of MSC biology that could potentially distort or inhibit the adoption of MSC-based therapies (Wilson et al., 2023). Central to this challenge is the need for precise clinical response prediction, which requires well-defined MSC populations and, contingent upon the therapeutic goal, an assessment of their desired specific activities such as differentiation potential, proliferation rate, secretory profile, and angiogenesis capacity.

### 3.1 Defining MSC subpopulations via specifically expressed surface antigens

It has been shown that ISCT criteria for phenotypic MSC identification can be insufficient for distinction between MSCs and certain other cell types, such as fibroblasts (Denu et al., 2016; Brinkhof et al., 2020; Budeus et al., 2023a). In fact, MSCs are morphologically indistinguishable from fibroblasts (Soundararajan and Kannan, 2018). What is more, Denu and colleagues have demonstrated that none of the ISCT criteria can reliably discern MSCs from fibroblasts, even arguing that they could represent the same cell type (Denu et al., 2016). Some suggest that fibroblasts could in certain instances be used as a more practical alternative to MSCs, while others maintain that they have complementary roles, especially in cell homeostasis and tissue development and injury (Ichim et al., 2018; Janja et al., 2021). Soundararajan and Kannan propose that the resemblance in characteristics between fibroblasts and aged MSCs, such as diminished differentiation potential, proliferation, immunomodulatory capacity, and specific cell surface markers, could mean that MSCs are in fact immature fibroblasts (Soundararajan and Kannan, 2018). Indeed, single-cell transcriptome sequencing has revealed that MSCs could constitute a subclass of fibroblasts (Fan et al., 2022).

On the other hand, gene expression and epigenetic studies have been successful in discerning MSC and fibroblast populations based on molecular signatures of homeobox genes and transcriptional factors (Taskiran and Karaosmanoglu, 2019; Budeus et al., 2023b). Likewise, comparative microarray transcriptome profiling of three fibroblast populations and MSCs from five different sources demonstrated a marked distinction between the “fibroblast” and “MSC” group, particularly in transcripts associated with structuration of the tissue skeleton (Haydont et al., 2020). Wiese and Braid propose a panel of 24 signature genes to support standardized and accessible MSC characterization, including five that have been shown to be upregulated in MSCs *versus* fibroblasts (Wiese and Braid, 2020).

So while RNA sequencing and microarrays could possibly discern between MSCs and fibroblasts, protocols for clinical application should be as straightforward and affordable as possible. Therefore the use of cell surface antigens would be preferable, however, to this date, they have proven insufficient to indisputably identify MSCs. Brinkhof et al. propose CD166 as a marker to differentiate MSCs from fibroblasts, as it is the only marker they have found to be upregulated in MSCs compared to fibroblasts. Notably, its expression levels positively correlated with those of CD105, although CD166 exhibited greater specificity for MSCs (Brinkhof et al., 2020). Nevertheless, Sober et al. did not detect significant difference in CD166 expression between MSCs derived from various tissues and fibroblasts. However, they put forward a panel of markers that could distinguish between MSCs originating from a specific tissue and fibroblasts; for instance, CD79a, CD105, CD106, CD146, and CD271 could be used to differentiate AD-MSCs from fibroblasts (Sober et al., 2023).

Adding to the complexity, a study on changes in MSC-related surface antigen expression during *in vitro* culture revealed that after 7 days, synovial fibroblasts began displaying MSC characteristics, further blurring the distinction between the 2 cell types (Isono et al., 2022).

Moreover, due to a multitude of discouraging clinical outcomes, there is a growing demand to identify additional surface markers capable of defining MSCs while capturing their biological and manufacturing variability, as well as clinical performance (Camilleri et al., 2016; Samsonraj et al., 2017). Consequently, various markers have been proposed to better define distinct subpopulations within MSCs (Smolinska et al., 2023).

Lately, CD146 has been frequently mentioned as a surface antigen that could serve as a marker for MSC potency evaluation (Bowles et al., 2020; Ma et al., 2021a). It is a transmembrane glycoprotein involved in adhesion, cellular signaling and numerous other physiological and pathological processes (Wang et al., 2020). It is expressed in MSCs derived from a wide range of tissue sources, both fetal and adult (Barilani et al., 2018). CD146 positive MSCs exhibit stronger proliferation, differentiation, migration and immunomodulatory abilities, however, prolonged passaging can result in progressive loss of CD146 on the cell surface (Yang et al., 2018; Al Bahrawy, 2021; Ma et al., 2021b; Zhang L. et al., 2022). Indeed, MSCs expressing high levels of CD146 display a more pronounced therapeutic effect compared to MSCs with low levels of CD146, as evident by increased survival rate in a mouse GvHD model (Bikorimana et al., 2022).

Another surface antigen implicated in MSC function is CD271, present on MSCs derived from adult but not fetal tissues. The reported abundance of CD271 varies widely, ranging from 4% to nearly 100%. Nevertheless, the majority of studies converge on an approximate 20% fraction of CD271+ positive cells within the bulk MSC population (Quirici et al., 2010; Watson et al., 2013; Beckenkamp et al., 2018; Smith et al., 2021). MSCs positive for CD271 display enhanced capacity for differentiation, proliferation, and colony formation when compared to their CD271− counterparts or mixed-population. However, the CD271 antigen diminishes rapidly with cell passaging, highlighting the importance of using cells subjected to minimal expansion procedures for therapeutic applications (Smith et al., 2021).

Moreover, CD271-depleted BM-MSCs have altered morphology, poor proliferation capacity, increased expression of hematopoietic markers, nearly no multi-lineage potential and are unable to form colonies, therefore failing to meet ISCT criteria for MSCs (Kuci et al., 2010; Petters et al., 2018). Notably, RNA sequencing analysis has revealed that there is a larger difference in gene expression between CD271+ and CD271− populations in AD-MSCs than between donors. Among genes with altered expression levels are those associated with inflammation and angiogenesis (Smith et al., 2021). MSC that are CD271 positive show superior promotion of cartilage repair to MSCs consisting of heterogeneous populations (Kohli et al., 2019). Nevertheless, when seeded on 3D osteoconductive biomaterial scaffold for bone regeneration, CD271+ MSCs proved inferior to heterogeneous MSCs, underscoring the need for caution when transferring results from monolayer to 3D cultures (Muller et al., 2019).

Several other surface antigens have been proposed as markers for MSCs and their specific subpopulations, such as STRO-1 as a marker for dental/gingival MSCs (Yu et al., 2010; Perczel-Kovách et al., 2021). Through advancements in methodologies, novel antigens are continually unveiled, prompting further investigations into their distribution and potential as phenotype markers. For example, based on single-cell RNA sequencing data, CD167b, CD91, CD130, and CD118 were proposed as novel surface markers for BM-MSC enrichment or purification. However, further molecular validations are needed (Wang et al., 2021b).

### 3.2 Functional assays

Contributing to the challenges in predicting clinical outcomes following MSC administration are the uncertainties surrounding mechanisms through which they drive tissue regeneration and exert immunoregulatory functions. Regulatory authorities require the development of tests to measure potency as part of release criteria of advanced clinical trials designed to support marketing approval and registration. Still, they allow for a considerable flexibility in determining the appropriate measurements of potency for each product and the adequacy of these tests is evaluated on a case-by-case basis (Galipeau et al., 2016). Ideally, the potency assay should reflect the *in vivo* mechanisms of action. However, since MSCs act through a complex range of mechanisms with unknown key steps, there are currently no definitive and unambiguous tests available that could accurately measure their clinical efficacy (Galipeau et al., 2016; Hematti, 2016; Robb et al., 2019).

Consequently, ISCT suggests an alternative approach. Rather than relying on a single potency assay, a collection of complementary assays should be conducted to evaluate relevant biological and therapeutic properties of the cells including quantitative analysis of mRNA expression, measurement of functionally relevant surface markers by flow cytometry, and protein-based assays to detect secreted factors (Galipeau et al., 2016).

#### 3.2.1 Differentiation potential of MSCs

One of the most intriguing and therapeutically promising MSC characteristics is their differentiation potential. Under both *in vivo* and *in vitro* stimulation, they can differentiate into several mesodermal-derived lineages, in particular chondrogenic, osteogenic, and adipogenic cells. Various studies suggest that they can also differentiate into non-mesodermal lineages like hepatocytes, neurons and pancreatic cells (Shibu et al., 2023). It is therefore important to distinguish MSC “potency” in a manufacturing context from their capacity to differentiate toward multiple cell lineages.

While tri-lineage differentiation potential is not typically assessed in MSC potency assays, it is still considered a key criterion for routine MSC characterization (Dominici et al., 2006; Montesinos et al., 2009). Therefore it is somewhat surprising, that most of the time researchers omit these assays, especially when conducting clinical studies (Wilson et al., 2021; Renesme et al., 2022). Furthermore, despite decades of undertaking MSC differentiation assays, there is still lack of consensus regarding the optimal media composition, detection reagents, and quantification methods to determine the extent of *in vitro* differentiation (Kakkar et al., 2020; Mollentze et al., 2021). Additionally, recent trends indicate a growing reliance on commercially available differentiation media, which are often elusive regarding their composition, thus exacerbating the heterogeneity among protocols (Eggerschwiler et al., 2019; Labedz-Maslowska et al., 2021; Bajetto et al., 2023).

When characterizing MSCs through tri-lineage differentiation in clinical and preclinical trials, researchers commonly present representative images of differentiated MSCs, confirmed through specific staining reagents (Aghayan et al., 2022; Shimizu et al., 2022; Krakenes et al., 2023). Following adipogenic differentiation, staining with Oil Red O or Nile Red is used to visualize intracellular lipid vacuoles. Osteogenic differentiation is confirmed by the detection of calcium deposits in the extracellular matrix via Von Kossa or Alizarin Red S staining. Chondrogenic differentiation is confirmed through the staining of cartilage deposits by Safranin-O/Fast Green or Alcian Blue (Ciuffreda et al., 2016).

However, when aiming to determine the extent of differentiation, colorimetric methods offer only semi-quantitative analysis options. Specifically, osteogenic differentiation can be evaluated by assessing extracellular calcium deposits through Alizarin Red S staining or by quantification of alkaline phosphatase activity (Widholz et al., 2019). Adipogenic differentiation can be estimated by staining intracellular lipid droplets with Oil Red O and chondrogenic differentiation by staining glycosaminoglycans, proteoglycans and collagen content with Safranin O. The extent of differentiation is then evaluated by semi-quantitative measurement of absorbance levels (Du et al., 2023).

Therefore, most of the studies that set to quantify the extent of MSC differentiation, have to analyze gene expression profile specific for the desired differentiation through real-time polymerase chain reaction (RT-PCR). Briefly, RNA extracted from cells that are undergoing chondrogenic, osteogenic, or adipogenic differentiation, is reversely transcribed, resulting in the generation of first-strand complementary DNA. This cDNA then serves as a template for amplification by RT-PCR using sequence-specific primers, followed by relative quantitation of gene expression (Kim et al., 2023). Certain changes in gene expression following MSC differentiation are well established, while others are still being discovered and could potentially serve as basis for quantification of differentiated MSCs (Hou et al., 2021; Stefanska et al., 2023).

In recent years, new methods for quantification of MSC differentiation are emerging. One of more compelling approaches is digital image analysis of histological staining. It aims to simplify the laboratory procedures to objectively quantify and classify the degree of differentiation as well as the differentiation potential among different MSC cell lines or cell subpopulations (Avercenc-Leger et al., 2017; Eggerschwiler et al., 2019). Another recently proposed strategy for determining the extent of osteogenic differentiation utilizes 18F. This radioactive tracer binds with a high affinity to newly synthesized hydroxyapatite and can then be evaluated by 18F μ-positron emission tomography scanning and activimeter analysis (Grossner et al., 2020). All in all, methods are continually being developed, in an effort to simplify and refine quantification of MSC differentiation and by extension, their therapeutic efficacy.

#### 3.2.2 Lymphocyte proliferation assay

While MSC ability to suppress lymphocyte proliferation is well documented, assays to quantify this process lack standardization and vary between groups and methodologies (Juhl et al., 2022). Still, *in vitro* inhibition of lymphocyte proliferation is considered a gold standard in predicting MSC functionality (Galipeau et al., 2016; Nicotra et al., 2020). Lymphocyte proliferation can be induced either by unspecific mitogens such as phytohaemagglutinin (PHA) and Staphylococcal enterotoxin B or by specific antibody-mediated activation of CD3 associated with the T cell receptor and CD28 for co-stimulatory signaling (Chinnadurai et al., 2018; Hammink et al., 2021). Alternatively, in mixed lymphocyte reactions (MLR) proliferation of T cells is activated by allorecognition, in which T cells are directly activated by allogeneic antigen-presenting cells (DeWolf et al., 2016). Lymphocyte proliferation can be quantified by several methods. Firstly, the amount of newly synthesized DNA can be measured by incorporation of thymidine analogs such as bromodeoxyuridine or 3H-thymidine (Svajger et al., 2021; Piede et al., 2023). Alternatively, total DNA content can be assessed by fluorescent dye binding using Hoechst or CyQUANT^®^ NF reagents (Suzdaltseva et al., 2022). One of the most widely accepted methods is tracking generations of cell division by dilution of covalently binding proliferation dyes such as CFSE (carboxyfluorescein succinimidyl ester) or CellTrace Violet (Kashef et al., 2022; Lemieszek et al., 2022).

However, these dyes can be toxic to a certain extent, prompting an exploration of alternative approaches. One such alternative is the assessment of cell proliferation by measuring the rate of metabolic activity. This can be achieved by utilizing colorimetric assays with tetrazolium salts or by quantifying the ATP levels with bioluminescent reagents that detect increases in healthy proliferating cells (Herzig et al., 2021; Herzig et al., 2023). Additionally, some researchers assess cell proliferation by measuring accumulation of cell cycle-associated proteins such as intracellular Ki67 or simply by counting cells, using, for example, CountBright Absolute Counting Beads (Cruz-Barrera et al., 2020).

In an effort to minimize the impact of donor variation, researchers pool peripheral blood mononuclear cells (PBMCs) from up to 10 different donors in MLR and mitogen induced lymphocyte proliferation (Christy et al., 2020). However, recent report indicates that optimal lymphocyte proliferation in MLR experiments can be achieved with as few as four donors (Hansen et al., 2022). Furthermore, two studies have identified PHA as the most suitable mitogen among tested components in their panels (Hansen et al., 2022; Kashef et al., 2022). It is noteworthy that, despite the conventional duration of 4–5 days for most lymphocyte proliferation assays, the peak of PBMC proliferation suppression occurs within 48 h following co-culture with MSCs, and extending the incubation window does not elicit significant changes (DeWolf et al., 2016; Suzdaltseva et al., 2022).

#### 3.2.3 Other assays

One of the assays to evaluate MSC potency, not required by ISCT but still frequently employed, is the endothelial cell tube formation assay. It evaluates MSC ability to support angiogenesis *in vitro* (Arnaoutova et al., 2009). Briefly, endothelial cells, whether primary or immortalized, are combined with MSC conditioned media and then seeded onto a basement membrane matrix. In response to angiogenic signals present in the media, cells start to rapidly form capillary-like structures. Within 1 hour, they align themselves, and by the second hour, tubules containing lumens begin to emerge. *In vitro* angiogenesis is then quantified as the number of branch sites/nodes, loops/meshes, or the number and length of tubes formed (DeCicco-Skinner et al., 2014; Carpentier et al., 2020). As the probable mechanism of action by which MSCs elicit an angiogenic response is through their paracrine activity, a quantification of expression of angiogenic factors and cytokines such as VEGF can be used to assess their angiogenic potency (Thej et al., 2017).

**FIGURE 3:**
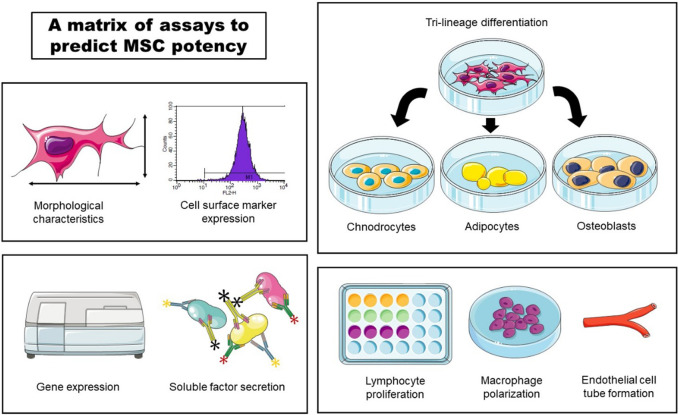
An example of a matrix of assays to predict MSC potency. Morphological characteristics, cell surface marker expression, gene expression, soluble factor secretion, tri-lineage differentiation, lymphocyte proliferation, macrophage polarization and endothelial cell tube formation could be used to assess potency of MSC cell product lots.

In accordance with ISCT guidelines, recent studies focus on developing assay matrix approach in order to predict MSC potency (Figure 3). One of the more commonly employed strategies involves utilizing secretory soluble factors, specifically chemokines and cytokines, through a multiplex analytical method (Lipat et al., 2022; Porter et al., 2022). Additionally, Kowal et al. claim that they could predict BM-MSCs proliferative capacity and the differentiation potential by assessing their morphological characteristics (Kowal et al., 2020).

In line with these efforts, Robb et al. developed an *in vitro* matrix of multivariate readouts, specifically, cell morphology, gene expression, soluble factor expression, macrophage polarization and angiogenesis (Robb et al., 2022b). Quantification of these critical quality attributes would serve to prospectively screen potent MSC donors or cell culture conditions to optimize for the desired basal MSC immunomodulatory or angiogenic fitness.

### 3.3 Autologous vs. allogeneic origin of MSCs

While autologous MSCs were historically favored for their lower risk of immune rejection, in recent years, the medical community has increasingly embraced allogeneic sources owing to immunological » invisibility« of MSCs in general. This shift can be ascribed to several factors, including their convenience, optimal donor and tissue selection, cost-effectiveness, as well as a compelling body of clinical evidence supporting their efficacy and safety. Still, some warn that the discrepancies between outcomes of murine preclinical models and human clinical trials could be attributed to the pre-clinical mouse data overwhelming use of syngeneic, major histocompatibility complex (MHC)-matched cells when examining efficacy endpoints (Galipeau and Sensebe, 2018; Giri and Galipeau, 2020). Indeed, repeated intra-articular injection of allogeneic mesenchymal stem cells in equine model resulted in an adverse clinical response, suggesting there is a potential for immune recognition of allogeneic MSCs upon repetitive exposures (Joswig et al., 2017).

Unfortunately, clinical trials directly comparing the application of allogeneic and autologous MSCs are rare and often inconclusive, primarily due to small sample sizes (Hare et al., 2012a). However, a study comparing safety and efficacy of autologous and allogeneic BM-MSCs in patients with non-ischemic dilated cardiomyopathy reported superior efficacy for allogeneic MSCs (Hare et al., 2012b)). A recent study comparing transplantation of autologous BM-MNCs (bone marrow mononuclear cells) to allogeneic WJ-MSCs into diabetic patients with chronic limb-threatening ischemia confirmed that both treatments are safe and effective. However, the therapeutic benefit was more pronounced when treating patients with allogeneic WJ-MSCs (Arango-Rodriguez et al., 2023). Furthermore, in a study investigating the potential of MSCs to alleviate GVHD following hematopoietic stem cell transplantation, there was no observed correlation between donor HLA-match and response rate (Le Blanc et al., 2008).

A recent review evaluating the outcomes of clinical trials using culture-expanded MSCs to treat osteoarthritis could not definitively distinguish between autologous and allogeneic MSCs in terms of efficacy (Copp et al., 2023). Similarly, *in vitro* study comparing immunomodulating effects of autologous and full HLA mismatched donor MSCs relative to the responder cells did not detect any significant difference in inhibition of PBMC proliferation (Waldner et al., 2018). Conversely, in a meta-analysis of randomized controlled trials comparing the efficacy and safety of autologous and allogeneic MSCs for knee osteoarthritis management, autologous MSCs emerged as superior in providing long-term pain relief and a lower incidence of adverse events (Jeyaraman et al., 2022).

Autologous MSCs sources can prove functionally inferior to allogeneic, especially when derived from donors with underlying systemic diseases. For example, AD-MSC derived from patients with chronic obstructive pulmonary disease exhibited decreased migration capacity than those derived from healthy donors. Still, they were equally efficient at reducing lung emphysematic damage in a mouse model (Rio et al., 2023). Importantly, autologous MSCs may exhibit suboptimal quality and fail to meet the requisite criteria for clinical utility, most often due to quality of starting material (Alves-Paiva et al., 2022). For example, a study investigating safety and feasibility of intramuscular transplantation of autologous BM-MSCs for patients with no-option critical limb ischemia reported a high rate of karyotype abnormalities in expanded cells (Mohamed et al., 2020). Moreover, MSCs derived from patients with type 2 diabetes displayed altered phenotype that most likely compromised their therapeutic efficacy (Capilla-Gonzalez et al., 2018). Similarly, MSCs derived from patients with systemic lupus erythematosus displayed dysfunctional phenotype and while patients responded to allogeneic MSC treatment, no response was seen with autologous cell transplantation (Cheng et al., 2019; El-Jawhari et al., 2021).

There is yet no clear clinical evidence to confirm whether autologous or allogeneic MSCs are superior to one another. It is plausible that specific conditions, especially those requiring tissue repair associated with MSC differentiation, may benefit more from autologous MSCs, while others, particularly may derive greater benefits from allogeneic MSCs. However, given the numerous advantages associated with allogeneic MSCs, particularly the greater ease of acquisition and production, it is probable that allogeneic MSCs will increasingly replace autologous cell sources.

## 4 Defining the optimal tissue source

Given the several tissues from which MSCs can be obtained, a natural question arises: which source is the most suitable? While multiple factors contribute to the decision made by institutions or medical teams, some sources offer more advantages than others. While bone marrow has traditionally served as a primary source for MSCs, the therapeutic potential of BM-MSCs is constrained by invasive harvesting techniques, suboptimal collection efficiency, age-related decline in quality, and donor-associated morbidities. On the other hand, a particularly advantageous source is the umbilical cord, primarily due to its noninvasive, low cost and ethically acceptable collection procedure.

### 4.1 Umbilical cord as a superior source of MSCs?

UC-MSCs can be isolated from various compartments including Wharton’s jelly, veins, arteries, umbilical cord lining, subamnion, perivascular regions, or the whole umbilical cord (Nagamura-Inoue and He, 2014). In recent years, Wharton’s jelly, the mucoid connective tissue surrounding the umbilical cord’s arteries and vein, has emerged as the preferred compartment for MSC isolation, although some argue that whole umbilical cord offers more advantages (Subramanian et al., 2015; Semenova et al., 2021).

UC-MSCs are considered more primitive than MSCs derived from adult tissues because they share more common gene expression with embryonic stem cells and show higher expandability *in vitro* (Hsieh et al., 2010; Musiał-Wysocka et al., 2019). Additionally, studies have reported a stronger immunomodulatory potential for UC-MSC in comparison to MSCs derived from alternative tissue sources. For example, higher expression levels of immunosuppressive molecules CD152 and HLA-G have been observed in UC-MSC compared to AD-MSC and DT-MSC (Zoehler et al., 2022). Furthermore, scRNA-seq data from MSCs originating from four distinct tissues reveal UC-MSCs as possessing the highest immunoregulatory scores among the analyzed samples (Hou et al., 2021). UC-MSCs have also demonstrated superior immunosuppressive function in comparison to BM-MSC in MLR and mitogen-induced T-cell proliferation (Ketterl et al., 2015b). Importantly, the use of UC-MSCs allows for planned selection of starting materials, using perfectly healthy donors, thereby avoiding potential functional compromises of final MSC products that could arise from patient-derived, autologous products (Oliva-Olivera et al., 2015; Widholz et al., 2019). It also allows for the opportunity to avoid age-related problems, which can represent an important issue BM-or AD-MSCs derived from elderly patients. Nevertheless, special attention should be given to gestational age when harvesting umbilical cords. As shown by Iwatani et al. UC-MSC proliferation can vary significantly when comparing pre-term and term UC-MSCs, a phenomenon demonstrated to be dependent by differential expression of the WNT pathway (Iwatani et al., 2017).

In clinical settings, BM-MSCs and UC-MSCs displayed comparable therapeutic effects when transplanted into patients with type 1 diabetes, including improvements in glycemic control and the preservation of β-cells (Zhang W. et al., 2022). More importantly, a systematic review investigating the generation of donor-specific antibodies after allogeneic MSC treatment has revealed that only when MSCs were obtained from the umbilical cord, there was no allo-response in any of the treated patients (Sanabria-de la Torre et al., 2021). The especially low immunogenicity of UC-MSCs compared to other MSC types renders their use particularly relevant in repetitive administration protocols, considering the possibility of immune sensitization and subsequent allo-rejection of the cellular product. Regarding specific aspects of their clinical utility, UC-MSC could prove to be superior in regenerative medicine particularly for treatments relying on increased neoangiogenesis. Namely, Kehl et al. have performed a detailed proteomic analysis, showing that MSC derived from wharton’s jelly display an enriched profile of angiogenic factors, with significantly higher concentrations of angiogenic proteins, compared to AD-MSCs and BM-MSCs (Kehl et al., 2019). Such differences between MSC types could have important implications for MSC selection in future clinical studies.

#### 4.1.1 Differentiation potential of UC-MSCs in tissue regeneration

Most of the studies report reduced differentiation capacity of UC-MSCs compared to MSCs derived from other tissue sources (Batsali et al., 2017; Todtenhaupt et al., 2023). It was suggested that the differential expression of the WNT pathway-associated molecules could have a role in the inferior osteogenic and adipogenic potential of UC-MSCs compared to BM-MSCs (Batsali et al., 2017). Interestingly, while UC-MSCs display a lower capacity for differentiation along osteogenic, adipogenic, and chondrogenic lineages compared to BM-MSCs, they outperform BM-MSCs in tenogenic differentiation. Namely, they exhibit superiority in forming a well-organized tendon-like matrix and in enhancing full-thickness tendon defect regeneration (Yea et al., 2023).

Along these lines, a meta-analysis comparing bone marrow aspirate concentrate (BM-AC) with CB-MSCs as a supporting treatment in various knee osteoarthritis patients undergoing high tibial osteotomy reported improved clinical outcomes in both groups. However, CB-MSCs allowed for a better articular cartilage regeneration than BM-AC augmentation (Park et al., 2023).

On the other hand, some studies have demonstrated higher osteogenic differentiation of UC-MSCs than BM-MSCs (Baksh et al., 2007). And a recent systematic review, centered on MSC transplantation for articular cartilage lesions in the human knee, established that UC-MSC transplants yielded superior outcomes when compared to BM-AC (Wang and Xing, 2023).

### 4.2 Pooled MSCs vs. MSCs from individual donors

The variability in biological properties among MSCs due to donor-to-donor heterogeneity is compromising the quality of data and hindering inter-study comparability. Pooling MSCs from several different donors has been proposed as a strategy to overcome these challenges (Zyrafete et al., 2016). The aim was to reduce the variance observed across donors. One of the first studies has shown that pooling MSCs leads to a greater increase of their ability to suppress lymphocyte proliferation than performances of MSCs derived from individual donors (Samuelsson et al., 2009). This was confirmed to some extent clinically by Ringden et al. where BM-MSCs from two different donors were pooled to treat a patient with myelofibrosis experiencing severe hemorrhage, yielding an encouraging outcome (Ringden and Leblanc, 2011).

Since then, several strategies of pooling MSCs have been tried, revealing interesting findings. More recently, we have also witnessed publications of GMP-compliant protocols for manufacture of MSCs, pooled from different donors (Padhiar et al., 2022). A study comparing the immunosuppressive potential of single batches to pooled products of MSCs prepared from iliac crest bone marrow aspirates did not show a significant difference that would favor pooled MSCs over MSCs from single donor batches. The allo-suppressive potential was comparable in both variants (Hejretova et al., 2020). Other studies comparing MSCs from various tissue sources have confirmed compensation for intra-individual variances among donors. However, there was no statistically significant difference in their immunosuppressive potential between the mean of a single MSC donor and pooled donors (Hansen et al., 2022). On the other hand, pooling BM-MNCs together before generation of BM-MSCs led to a significantly higher allosuppressive potential of the pooled cells. This phenomenon indicates that MNC pooling induces a strong alloreaction, which could positively select progenitor cell fractions for MSCs with higher allosuppressive potential, either through cell–cell interactions and/or soluble molecules (Zyrafete et al., 2016; Bieback et al., 2019). Among the published data, Waldner et al. had a surprisingly different approach–they pooled cells from the same donor but from different tissue sources, specifically bone marrow and adipose tissue. Interestingly, the pooled cells demonstrated a synergistic immunosuppressive effect on PBMC proliferation (Waldner et al., 2018).

Examining other characteristics after establishing MSC pools reveals varied reports. Some studies confirmed a correlation between mean proliferation rate of individual donors to proliferation rate of pooled MSCs (Zyrafete et al., 2016). Others, however, observed a higher population doubling for pooled cells compared to individual batches, inferring that the fast proliferating cells contribute more towards the whole cell population in the pooled setting, resulting in faster overall proliferation rate and eventually increasing the relative portion of the respective cells (Widholz et al., 2019). It has been shown that pooling BM-MSCs at different passages does not change their functional characteristics or diminish their quality (Willer et al., 2022). Additionally, introducing multiple rounds of cryopreservation of pooled cells does not induce significant changes in their fundamental characteristics (Mamidi et al., 2012). However, comparing WJ-MSCs from individual donors to cells pooled from three donors revealed lower levels of expression of pro-inflammatory cytokines in the pooled batches. This observation prompted speculation that the act of pooling MSCs could potentially create a slightly inflammatory microenvironment, consequently directing MSCs toward a more immunosuppressive phenotype. Still, the immunosuppressive potential of the pooled MSCs remained similar to that of individual donor cells (Kannan et al., 2022).

**FIGURE 4:**
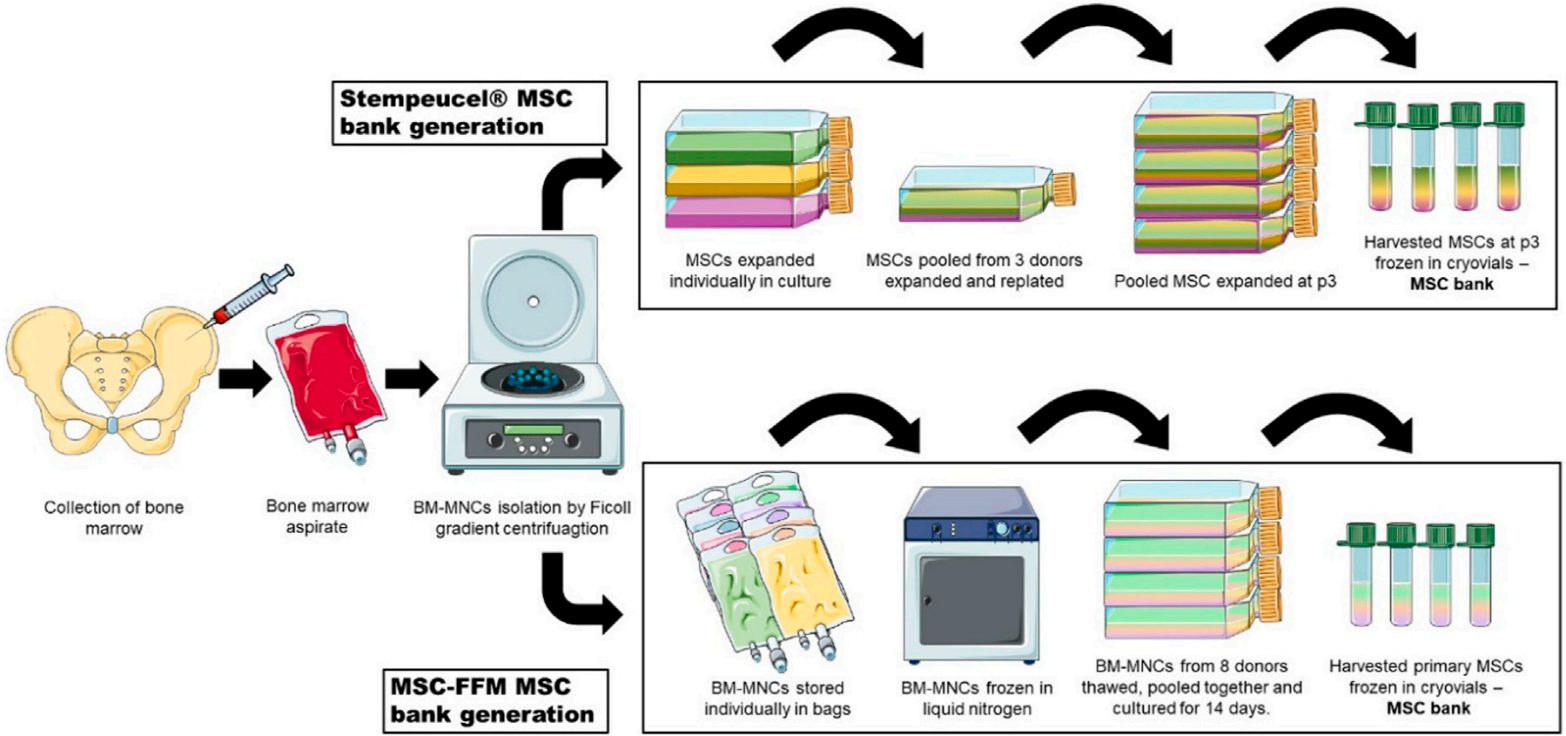
Two strategies of MSC pooling currently used in therapeutic applications. MSCs from three donors are individually expanded and then pooled together for Stempeucel^®^ MSC bank generation. On the other hand, in MSC-FFM, MNCs from eight donors are pooled together before cells attach to the surface of tissue culture flasks. After expansion, cells are harvested and stored in cryovials to constitute a MSC bank.

Currently, two MSC products containing pooled cells for therapeutic applications are in active use (Figure 4). Leading the way is Stempeutics, an India-based company and the first to commercialize pooled MSCs. Their advanced therapy medicinal product, Stempeucel^®^, consists of BM-MSCs pooled from three healthy donors and is intended to treat a variety of conditions such as limb ischemia, osteoarthritis of the knee joint, perianal fistulas and diabetic foot ulcers (Rengasamy et al., 2016; Gupta et al., 2017; Thej et al., 2017; Gupta et al., 2023a; Gupta et al., 2023b). The other is generated from pooled BM-MNCs from eight allogeneic donors, also referred to as “MSC-Frankfurt am Main” or MSC-FFM, named after the city where it was produced. This pooled product is licensed with a national hospital exemption authorization in Germany. It is applied to patients with steroid and therapy-refractory acute GvHD and was shown to be safe and effective (Zyrafete et al., 2016; Bader et al., 2018; Bonig et al., 2019).

While the use of pooled MSCs definitely represents several potential advancements toward product harmonization, as well as offering several logistical solutions, potential limitations should be addressed. One such is the potential for alloreactive immune response associated with allogeneic cell therapy treatments. Most likely, this is not an issue of great concern, since MSCs are widely recognized as hypoimmunogenic (due to low expression of HLA molecules) and their allogeneic clinical use is extensively documented, confirming safety. Nevertheless, consideration of histocompatibility barriers along with anticipation of immune rejections and immune sensitization reactions should be kept in mind. For example, certain percentage of patients have been shown to develop alloantibodies and subsequent immune rejection of administered MSCs (Sanabria-de la Torre et al., 2021). Even in case of UC-MSCs, where the formation of alloantibodies is seldom reported, this phenomenon could be potentially amplified using pooled MSC products from different donors, where HLA diversity is further increased.

## 5 Conclusion

The potential of MSCs to regulate the host immune system and promote tissue regeneration through paracrine signaling offers a great promise for addressing a variety of issues. However, studies that would reproducibly and reliably confirm this potential in clinical setting are still lacking. One of the culprits most frequently implicated in these discrepancies is the heterogeneity of transplanted MSC batches. It can arise from inherent biological differences among tissue sources, donors and MSC subpopulations or it can be introduced by variations in preparation protocols. Strategies to mitigate these differences could range from careful selection of tissue source, donors and specific MSC subpopulations, to harmonized growing conditions, potency assays and administration protocols. Amidst the multitude of options, we also propose an off the shelf approach of pooling UC-MSCs to increase consistency and homogeneity of the final cell product. UC-MSCs are relatively easy to obtain and have several advantageous characteristics in comparison to MSCs derived from other tissue sources. Moreover, pooling UC-MSCs from several donors would reduce inter-donor variability, improve dose-to-dose equivalence between patients, and facilitate the comparison of therapeutic efficacy across clinical studies. This approach could be relatively easily implemented in hospital GMP manufacturing centers. The main pre-requisites are established standard operating protocols in association with maternity wards for the supply of biological starting materials, and obviously for MSC manufacture. In addition, the pre-requisite that a well-managed cryobank is present for storing allogeneic MSC aliquots is key. In closing, we propose increased academic research efforts in the area of MSC pooling, to further resolve potential benefits, as well as its limitations and challenges on the path toward standardized and homogeneous MSC-based ATMPs.
